# DEVELOPING SOCIAL CONVOY PALLIATIVE CARE MOBILE HEALTH FOR OLDER ADULTS

**DOI:** 10.1093/geroni/igz038.721

**Published:** 2019-11-08

**Authors:** Jennifer D Portz

**Affiliations:** University of Colorado, Aurora, Colorado, United States

## Abstract

Social Convoy Theory is well established in the gerontology literature and provides a framework for understanding the relationships of individuals in a group of people with whom they give and receive social support over the life-cycle. Previous research substantiates a link between the importance of social convoys and health outcomes among older adults with advanced illness. However, the concept of social convoy has not been applied to mobile health (mHealth) for palliative care. Often mHealth systems are designed for individual users, lacking innovative solutions for multiple, age-diverse, simultaneous users to maximize benefit. Therefore, the Social Convoy Palliative Care (Convoy-Pal) mobile application (K76AG059934) was developed using a rigorous iterative process with older patients, their social convoy, and health providers, to foster effective integration of the social convoy in palliative care-specific mHealth. Based on these findings, this presentation discusses strategies to design mHealth for a convoy of users in geriatric palliative care.

